# Comparative analysis of wild and cultivated Lathyrus L. species to assess their content of sugars, polyols, free fatty acids, and phytosterols

**DOI:** 10.18699/VJ20.667

**Published:** 2020-11

**Authors:** A.E. Solovyeva, T.V. Shelenga, A.L. Shavarda, M.O. Burlyaeva

**Affiliations:** Federal Research Center the N.I. Vavilov All-Russian Institute of Plant Genetic Resources (VIR), St. Petersburg, Russia; Federal Research Center the N.I. Vavilov All-Russian Institute of Plant Genetic Resources (VIR), St. Petersburg, Russia; Federal Research Center the N.I. Vavilov All-Russian Institute of Plant Genetic Resources (VIR), St. Petersburg, Russia St. Petersburg State University, St. Petersburg, Russia V.L. Komarov Botanical Institute of the Russian Academy of Sciences, St. Petersburg, Russia; Federal Research Center the N.I. Vavilov All-Russian Institute of Plant Genetic Resources (VIR), St. Petersburg, Russia

**Keywords:** Lathyrus L., wild species, varieties, green mass, gas chromatography, polymorphism of characters, Lathyrus L., дикие виды, сорта, зеленая масса, газовая хроматография, генетические ресурсы, полиморфизм признаков

## Abstract

Under the condition of climate change, the need for crops resistant to abiotic and biotic stresses is increasing. Lathyrus spp. are characterized by a high nutritional value of their green biomass. The grass pea is one of the most resistant to drought, waterlogging, cold, salinity, diseases and pests among cultivated legumes, and it is grown at minimal cost. The creation of new Lathyrus L. sorts with an improved nutrient composition of nutrients will allow to obtain high-quality feed in areas with extremely unstable weather conditions. In this connection, we studied the patterns of variability in the parameters of the carbohydrate complex (sugars, their lactone and methyl forms), polyols (including phenol-containing alcohols), phytosterols, free fatty acids (FFA) and acylglycerols in the green mass of 32 samples of Lathyrus sativus L., L. tuberosus L., L. sylvestris L., L. vernus (L.) Bernh., L. latifolius L., L. linifolius (Reichard) Bassler. from the VIR collection, reproduced in the Leningrad region in contrasting conditions 2012, 2013.The content of identified compounds varied depending on the genotype, species, and weather conditions. High temperatures and high level of precipitation in 2013 contributed to the accumulation of monosaccharides, in more colder and drier conditions in 2012 – oligosaccharides, most of polyols and FFA. The cultivated species (L. sativus) was distinguished by its high sugar content, and the wild species as follows: L. latifolius by FFA; L. linifolius by ononitol, myo-inositol, and glycerol 3-phosphate; L. vernus by MAG and methylpentofuranoside. The species cultivated in culture (L. sativus) was distinguished by a high sugar content, wild species: L. latifolius – by FFA, L. linifolius – ononitol, myo-inositol and glycerol-3-phosphate, L. vernus – MAG and methylpentofuranoside. According to our results, the studied samples are promising for the selection of Lathyrus varieties with high nutrition quality and stress-resistant.

## Abstract (ru)

В условиях изменения климата увеличивается потребность в устойчивых к абиотическим и биотическим стрессам сельскохозяйственных культурах. Виды чины характеризуются высокой питательной ценностью зеленой массы. Чина посевная – одна из наиболее устойчивых к засухе, заболачиванию, холоду, засолению, болезням и вредителям бобовых культур, выращивается при минимальных затратах. Создание сортов Lathyrus L. с улучшенным составом питательных веществ позволит получать качественные корма в районах с крайне неустойчивыми погодными условиями. В связи с этим нами исследованы закономерности изменчивости показателей углеводного комплекса (сахаров, их лактонных и метильных форм), многоатом- ных спиртов (в том числе фенолсодержащих), фитостеролов, свободных жирных кислот и ацилглицеролов в зеленой массе 32 образцов Lathyrus sativus L., L. tuberosus L., L. sylvestris L., L. vernus (L.) Bernh., L. latifolius L., L. linifolius (Reichard) Bassler. из коллекции ВИР, репродуцированных в Ленинградской области в контрастных условиях 2012 и 2013 гг. Содержание идентифицированных соединений варьировало в зависимости от генотипа, вида и метеорологических условий. Высокие температуры и большое количество осадков 2013 г. способствовали накоплению моносахаридов, более холодные и сухие условия 2012 г. – накоплению олигоса- харидов, большинства многоатомных спиртов и свободных жирных кислот. Вид, возделываемый в культуре (L. sativus), отличался высоким содержанием сахаров, дикие виды – свободных жирных кислот (L. latifolius), ононитола, мио-инозитола и глицерол-3-фосфата (L. linifolius), МАГ и метилпентофуранозида (L. vernus). По ре- зультатам изучения большинство исследованных образцов перспективно для селекции новых высокопита- тельных и устойчивых к стрессам кормовых сортов Lathyrus.

## Introduction

The changing climate leads to the expansion of areas with extremely unstable weather conditions, thus enhancing the demand for stress-resistant crops grown for food and feed. Many species in the genus Lathyrus L. are used as sources of human food, animal feed, and medications. A majority of wild peavines are exploited as pasture and fodder plants. The best-known species is the grass pea (Lathyrus sativus L.) with its millennia-long cultivation history, cultivated on all the continents. This leguminous crop is considered one of the most resistant to drought, waterlogging, and cold (Campbell, 1997). It is adapted to a diversity of soil types, including salinized soils, and would yield harvests in the environments where other crops would die, so it was recognized as ‘the food for survival’ (Sarkar et al., 2019). The species is resistant to diseases (powdery mildew, rust, etc.) and pests (Sarkar et al., 2019). The yield of grass pea seed reaches 2.9 t/ha, and that of its green biomass is 5.2 t/ha.

Grass pea seed and green biomass are notable for their high nutritional value. The grain contains, depending on growing conditions, from 18 to 34 % of protein (Rizvi et al., 2016; Burlyaeva et al., 2018; Donskoj et al., 2019), and the biomass from 10 to 27 % (Burlyaeva et al., 2015). Seeds of L. sativus and L. cicera L. are characterized by high concentrations of essential amino acids (63–64 %) and polyunsaturated fatty acids (66.9 and 58.6 %, respectively), predominantly linoleic (Grela et al., 2012). The hay from grass pea matches alfalfa in nutritiousness (Poland et al., 2003). The content of organic acids in the Lathyrus green biomass varies from 140.0 to 2140.0 mg/100 g, free amino acids from 11.8 to 610.0 mg/100 g, and secondary metabolites from 4.4 to 224.6 mg/100 g (Solovyeva et al., 2019).

In Russia, compared with other countries, Lathyrus spp. occupy a minor niche in the national plant production. Breeding work is conducted in a limited number of institutions, which has a negative effect on the crop’s utilization in agriculture.

Our previous investigations of the green biomass of wild and cultivated Lathyrus spp. exposed a wide range of compounds in it (organic acids, free amino acids, and secondary metabolites). Accessions from the peavine collection were identified as promising sources for the development of highly nutritious, resistant and officinal cultivars (Solovyeva et al., 2019).

The present study is the next step in the research into metabolomic profiles of peavine green biomass with a focus on the content of saccharides, free fatty acids (FFA), polyols, and acylglycerols. The objective of the study was to assess the interspecific and intraspecific polymorphisms of biochemical characters in Lathyrus spp. and the effect of weather conditions on their variability. To solve this task, the technique currently widely known as profiling was used (Steinhauser, Kopka, 2007). This technique is one of the so-called metabolomic methods of analysis, which, in addition to delivering information on individual metabolites in a studied object, makes it possible to evaluate the status of this object (Worley, Powers, 2012; Hong et al., 2016).

Thus, our aim was gaining knowledge about variations in the status of metabolic networks in the accessions of Lathyrus spp. in the context of their taxonomic characteristics and weather conditions.

## Materials and methods

The experiment encompassed 32 accessions of six Lathyrus spp. from the collection of the Vavilov Institute (VIR): grass pea (L. sativus), flat pea (L. sylvestris L.), spring pea (L. vernus (L.) Bernh.), heath pea (L. linifolius (Reichard) Bassler), everlasting pea (L. latifolius L.), and tuberous pea (L. tuberosus L.), grown in the fields of Pushkin experimental laboratories of VIR in 2012 and 2013 according to the guidelines approved by VIR (Vishnyakova et al., 2010). Meteorological conditions during the growing seasons were contrasting. In 2012, the sum of active temperatures was 1885.0 °С, and the precipitation amount 340.7 mm; in 2013, the sum of active temperatures increased to 2474.3 °С, and rainfall to 646.4 mm.

Plants were collected in the phase of first mature pods. Fresh green biomass was analyzed from 5 plants of each accession (leaves, inflorescences, pods in the phase of milk ripeness, and stems). Gas chromatography/mass spectrometry (GC–MS) profiling was performed using the protocol for the analysis of trimethylsilyl derivatives, developed at the Komarov Botanical Institute while working on the program task, theme АААА-А18-118032390136-5 “Assessment of changes in the correlational structure of metabolite networks in the process of growth and development of fungi and plants from the viewpoint of systemic biology”, and adjusted for use on Agilent 6850-MSD 5975 at the Research Park of St. Petersburg State University Center for Molecular and Cell Technologies (Puzanskiy et al., 2018).

The plant material was examined by the GC–MS technique: it was extracted with ethanol, and then evaporated dry on a CentriVap Concentrator (Labconco, USA). The solid residue was dissolved in pyridine containing 1000 ppm of tricosane that served as an internal standard; then, 20 μl of BSTFA (N,O-Bis[trimethylsilyl]trifluoroacetamide) (Supelco, USA) was introduced. To ensure the silylation reaction sufficiency, the vials were kept for 15 min under +100 °С in a special thermal block. The samples were analyzed on an Agilent 6850 chromatographic mass spectrometer with the Agilent 5975 D mass selective detector (USA). Chromatographic separation was done on an Agilent HP-5MS column (USA), length: 30 m, internal diameter: 0.25 mm, stationary phase film thickness: 0.25 μm, linear temperature programming mode: 70 to 325 °C, speed: 6°/min (50 min), carrier gas: helium. The analysis was performed with a constant gas flow velocity through the column (1 mL/min) as follows: evaporator temperature: +300 °С; flow split ratio during sample injection: 1: 20; mass spectra scanning range: 50 to 1050 amu; scanning speed: 2 scans/sec. Total ion current (TIC) chromatograms were recorded for the samples.

The results were processed using UniChrom and AMDIS software resources, NIST 2010 mass spectra libraries, and in-house libraries of the Research Park of St. Petersburg State University and the Komarov Botanical Institute.

The amount of trimethylsilyl (TMS) derivatives of the identified compounds was calculated by the internal standardization method for tricosane using UniChrom software. With the semiquantitation approach applied, the detector’s sensitivity coefficients for individual compounds need not be taken into account (Worley, Powers, 2012). The data produced by the analysis are presented in conventional units (c. u.) (Sitkin et al., 2013).

Statistical data processing was done with Statistica 7 and Exсel 7.0 software for Windows, using the principal factor analysis (PFA) and one-way analysis of variance (ANOVA). Statistical significance of the environmental effect on the expression of biochemical characters was assessed using Fisher’s criterion (LSD test), and the factor’s effect size (percentage) – η^2^ (intraclass correlation coefficient, according to Fisher) – was calculated by the following formula (Ivanter, Korosov, 2003):

**Formula:**
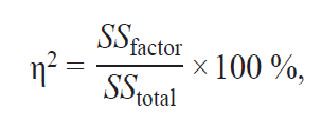
1

where η^2^, % is the effect size percentage of the factor’s impact; SS_factor_ is the sum of squared deviations for the factor; SS_total_ is the total sum of squared deviations.

## Results

The GC–MS profiling of the green biomass of Lathyrus L. spp. identified about 300 components. Organic acids, free amino acids and phenol-containing compounds were discussed in an earlier publication (Solovyeva et al., 2019). This study presents analytical results of comparing the contents of over 60 compounds in the peavine green biomass, including such groups as sugars, polyols, phytosterols, and FFA. The levels of the identified compounds are presented in c. u. (see the Table, Suplementary Material 1)^1^.

Supplementary Materials are available in the online version of the paper: http://www.bionet.nsc.ru/vogis/download/pict-2020-24/appx11.pdf



**Fig. 1.**
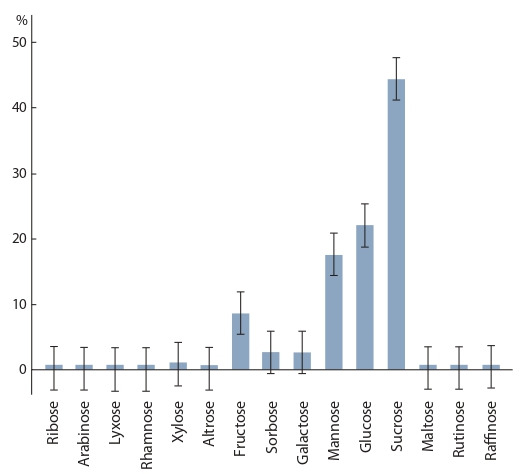
Component composition profiles of monosaccharides and oligosaccharides: percentage of the total sugar content in the green biomass of Lathyrus spp.

**Carbohydrate composition.** Sugars in the peavine green biomass consisted of mono- and oligosaccharides. Monosaccharides were represented by pentoses (ribose, arabinose, lyxose, and xylose) and hexoses (fructose, glucose, sorbose, galactose, mannose, rhamnose, and altrose). Oligosaccharides included disaccharides (sucrose, maltose, and rutinose) and a trisaccharide (raffinose). Fructose, glucose and mannose were the main sugars in the hexose group, xylose in the pentose group, and sucrose in the group of disaccharides (Fig. 1). Besides, metabolically active forms of saccharides were idenified: lactone (glucose 1,4-lactone), phosphate (fructose 6-phosphate and glucose 1-phosphate), and methyl sugars (methylpentofuranoside and methylglucofuranoside).

**Table 1.**
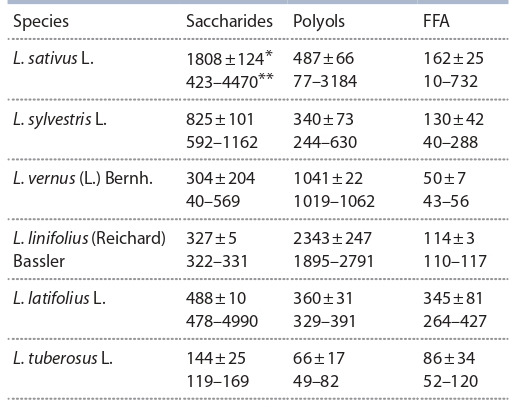
Comparative analysis of the content of saccharides, polyols and FFA in the green mass of some Lathyrus L. species (c. u.) Arithmetic mean ± standard error of the arithmetic mean; ** variability (min–max).

**Fig. 2.**
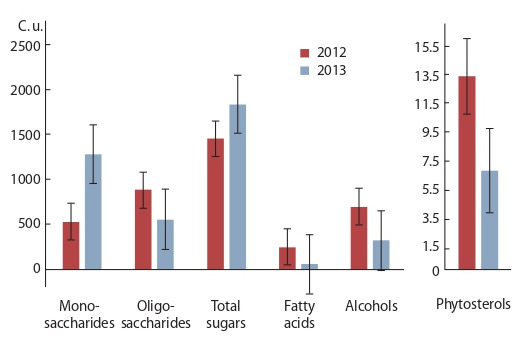
Mean values for monosaccharides, oligosaccharides, total sugars, fatty acids, polyols, and phytosterols in the Lathyrus green biomass in 2012–2013.

The relative content of sugars varied across the years (Fig. 2) and the genotypes. A majority of sugars were monosaccharides (67 % of the total sugars) represented mostly by hexoses (66 % of the total sugars). Pentoses amounted to slightly more than 1 %, and oligosaccharides to 32.6 %; of the latter, disaccharides accounted for 32.3 %, and trisaccharides for 0.3 %. Sucrose had the highest percentage among disaccharides (31.9 %).

The mean value for sugars in 2012 was 1442 c. u. (40 to 4470). The greatest part of sugars was represented by disaccharides, their mean content equal to 903 c. u. (0 to 3983). The mean total amount of monosaccharides was 538 c. u. (40 to 2085). They were represented mostly by hexoses, with the mean level of 530 c. u. (29 to 2082). Pentoses averaged 8 c. u. (0 to 24). Identified among trisaccharides was raffinose whose mean content was 1 c. u. (0 to 14). The mean content of total oligosaccharides was 904 c. u. (0 to 3984).

In 2012, the highest saccharide levels were recorded for the accessions of L. sativus (up to 4470 c. u.) and L. sylvestris (up to 1162). The remaining peavine species accumulated less than 600 c. u. of sugars. The lowest values were observed in the accessions of L. tuberosus (144 c. u.).

In 2013, the total content of sugars, including monosaccharides (hexoses and pentoses), increased on average to 1830 and 1278 c. u., respectively, while the content of sucrose reduced to 551. Compared with 2012, the limits of the variation range changed for total sugars from 144 to 2511 c. u., for monosaccharides from 63 to 1842 c. u. (for hexoses from 63 to 1751 c. u.), and for oligosaccharides from 273 to 1060 c. u. The major part of sugars in 2013 was represented by monosaccharides (hexoses). Mean values for total oligosaccharides in 2013 decreased to 532 c. u., while those for raffinose increased to 11 c. u. The ranges of variability for pentoses and raffinose (0.3–64 and 0–40 c. u., respectively) in 2013 became wider than in the previous year.

High levels of sugars (more than 2831 c. u.) during the entire period of studies were observed in the accessions of L. sativus.

**Polyols and phytosterols.** Most of the identified polyols were hexatomic (sorbitol, dulcitol, mannitol and inositol); their mean total amount was 476 c. u. (see Suppl. Material 1). They were represented mainly by inositol, its isomers and derivatives (myo-inositol, chiro-inositol, methyl-inositol, ononitol and galactinol); their total sum was 413 u. e. In addition to the alcohols mentioned above, glycerol, erythritol, threitol, xylitol, arabinitol and phytol were identified. Besides, phytosterols (campesterol, stigmasterol, β-sitosterol, isofucosterol and taraxasterol) and phenolic alcohols (coniferol, α-tocoferol, kaempferol and pyrogallol) were observed; their total sums were 9 and 3 c. u., respectively. In the phytosterol group, β-sitosterol prevailed (8 c. u.). Phosphate forms of glycerol and inositol were also identified as well as metabolic products of glycerophospholipids (glycerol 3-phosphate and myo-inositol 2-phosphate). Phenol-containing alcohols were discussed earlier (Solovyeva et al., 2019).

The content of polyols significantly varied across the years of the study (see Fig. 2). In 2012, their mean level was 744 c. u., with the limits of variation from 171 to 3184. In 2013, their amount reduced to 317 c. u., and the range of their variation narrowed (77 to 442). This value in L. sativus in 2012 (637 c. u.) was lower than the mean (calculated for all accessions), but in 2013 it decreased to 322 c. u. The same tendency was observed in the accessions of L. sylvestris: 437 c. u. in 2012, and 275 c. u. in 2013.

The highest content of polyols was found in L. sativus (3152 c. u.), a lower one in the accessions L. linifolius (2307), L. vernus (1050), L. sylvestris (619) and L. latifolius (348), and the lowest in L. tuberosus (66).

The highest amounts of erythritol and phytosterols (1 and 22, respectively) were observed in the accessions of L. sativus, and the highest total polyols in L. sativus and L. linifolius (3115 and 2307 c. u., respectively).

**Free fatty acids and acylglycerols.** Nineteen FFA were identified, including saturated (capric, undecylic, lauric, palmitic, stearic, arachidic, behenic, lignoceric, cerotic, montanic and melissic) and unsaturated ones (oleic, linoleic and linolenic), hydroxy acids (hydroxyoctadecanoic, hydroxytetracosanoic, hydroxyhexacosanoic, hydroxyoctacosanoic and hydroxytriacontanoic), and monoacylglycerols (MAG-1 C16:0 and MAG-1 C18:0) (see Suppl. Material 1).

In 2012, the total FFA content in the peavine green biomass was 248 c. u. (limits of variation from 37 to 732), with 1 c. u. for MAG (0 to 21). In 2013, the values of FFA and MAG reduced to 52 and 0.5 c. u., and their variation limits narrowed (10 to 138, and 0 to 2, respectively) (see Fig. 2).

Hydroxyhexacosanoic, palmitic, linolenic and stearic acids showed the highest values: their shares in the total content of FFA and hydroxy acids were 32, 22, 14 and 13 %, respectively. The mean percentage of linoleic acids was 7 %, with 5 % of capric, 3 % of oleic, 2 % of hydroxyoctacosanoic, and 1 % of undecylic acid. The percentage of minor FFA was less than 1 %.

The highest mean content of FFA was observed in the accessions of L. latifolius (up to 345 c. u.). Other species showed lower levels: 162 c. u. in L. sativus, 131 in L. sylvestris, 114 in L. linifolius, and 86 in L. tuberosus. The lowest FFA amounts were found in L. vernus (50 c. u.), but this species was distinguished for the contents of MAG-1 C16:0 (up to 11 c. u.) and MAG-1 C18:0 (up to 10). The levels of MAG in the other species were much lower.

## Discussion

Our experiment disclosed the presence of significant interspecific and intraspecific variability of the Lathyrus accessions both in the quantitative content and qualitative composition of the identified compounds (see Suppl. Material 1).

The analysis of the resulting data showed a strong variation in the values under different weather conditions (see Fig. 2). High temperatures and intense rainfall (2013) contributed to the accumulation of sugars at the expense of an increase in the percentage of monosaccharides, while the colder and drier conditions (2012) provoked a rise of polyols, FFA and oligosaccharides.

**Fig. 3.**
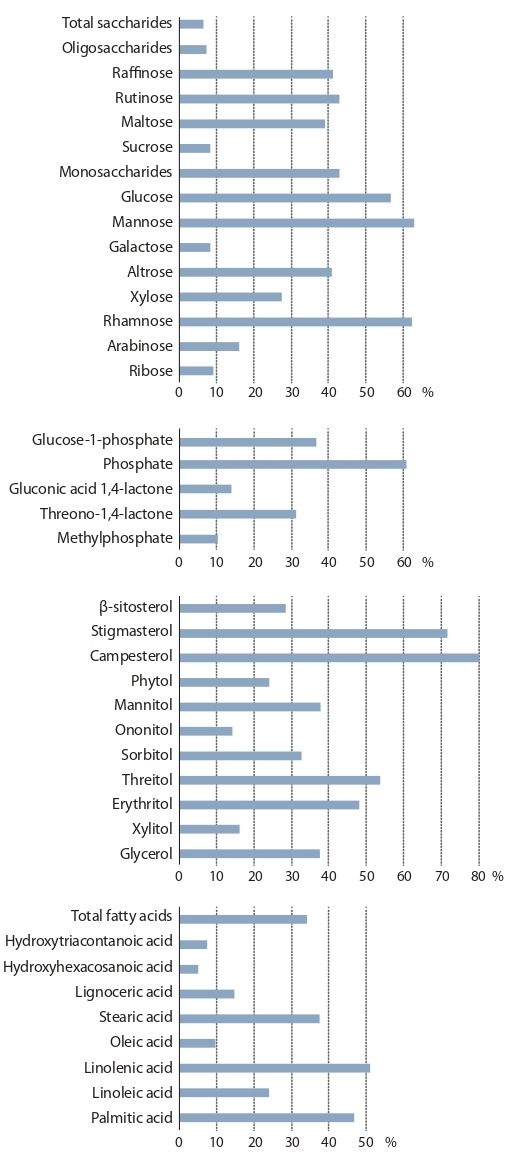
The effect size percentage (η2) showing the impact of weather conditions on the variability of biochemical characteristics.

A single factor analysis of variance was applied to ascertain the statistical significance of the effect of weather on the studied characters. Growing conditions were found to produce a statistically significant impact on the variability of the total sugars, total FFA, ribose, arabinose, xylose, altrose, rhamnose, mannose, glucose, galactose, sucrose, maltose, rutinose, raffinose, certain acids (palmitic, stearic, oleic, linoleic, linolenic, lignoceric, hydroxyhexacosanoic and hydroxytriacontanoic), stigmasterol, threitol, glycerol, xylitol, erythritol, ononitol, sorbitol, mannitol, phytol, β-sitosterol, campesterol, phosphate, glucose 1-phosphate, methylphosphate, threono-1,4-lactone, and glucono-1,4-lactone (Fig. 3, Suppl. Material 2). Variations of other compounds were not significantly affected by weather. The strongest effect of growing conditions was observed in the accumulation of mannose (effect size η2 = 62.9 %), rhamnose (62.3), glucose (56.7), raffinose (41.1) and altrose (40.9 %) in the peavine biomass. Variability of the total FFA levels was determined by meteorological conditions to the extent of 34.1 %; linolenic (51.1) and palmitic (46.9) acids were the most dependent on them. Among phytosterols, the highest values of η2 were recorded for campesterol (80.2 %) and stigmasterol (71.6); among polyols, for threitol (53.6 %) and mannitol (37.9). A significant weather impact was produced on the content of phosphoric acid derivatives (60.0 %), glucose 1-phosphate (36.7), and threono-1,4-lactone (31.5). Thus, weather conditions were found to have a significant effect on the accumulation of a considerable number of identified compounds in the green biomass of Lathyrus spp.

The principal factor analysis (PFA) was applied to reveal the interplay among biochemical characters and disclose regularities in their variations under the influence of weather conditions, genotypes, and taxonomic (species-specific) attribution. As a result, 10 factors determining 65.8 % of the total variance in plant characters were identified. In the first factor (F1, 20.7 % of the total variance), two large groups of biochemical characters demonstrated negatively correlated concurrent variations. The first group included rhamnose, xylose, altrose, glucose, maltose, rutinose, raffinose, erythritol, threitol, mannitol, campesterol, stigmasterol, phosphate and glucose 1-phosphate; the second one united palmitic, linoleic, linolenic and stearic acids, glycerol, sorbitol, β-sitosterol and threono-1,4-lactone. This factor demonstrates that higher amounts of the compounds from the first group in peavine green biomass are accompanied by lower levels of those from the second group, and vice versa. The dominating characters in F1 (determining variations of the others) are campesterol, stigmasterol, linolenic acid, and palmitic acid. The second factor (F2, 7.5 %) proves the relationship between sorbose and fructose. The third (F3, 6.8 %) ascertains the interactions of methyl-inositol, glyceraldehide, α-methylglucofuranoside, and methylglucoside. The fourth (F4, 6.3 %) shows correlations between capric, oleic and hydroxytetracosanoic acids. The fifth (F5, 5.4 %) incorporates arachidic, behenic, hydroxyhexacosanoic and hydroxyoctacosanoic acids. The sixth factor (F6, 4.8 %) includes melissic and hydroxyoctadecanoic acids, the seventh (F7, 4.1 %) ononitol, the eighth (F8, 3.6 %) glycerol 3-phosphate, the ninth (F9, 3.5 %) lupeol and guanosine, and the tenth (F10, 3.3 %) myo-inositol 2-phosphate and uridine.

**Fig. 4.**
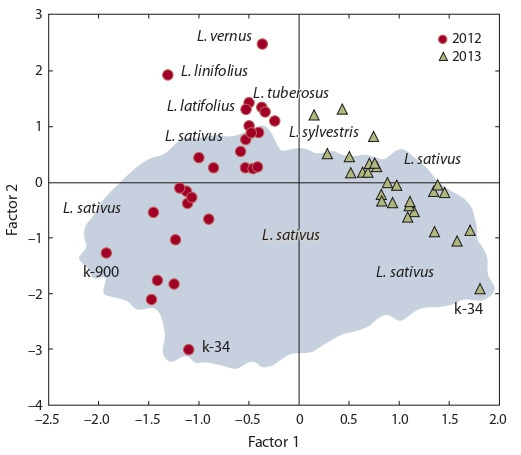
Distribution of the accessions over the space of the first two factors (F1 and F2).

While studying the distribution of accessions in the space of the first two factors, it seems obvious that the plants are grouped according to the years of observations (Fig. 4). The right-hand part of the graph contains cultivars with high levels of saccharides (rhamnose, xylose, altrose, glucose, maltose, rutinose, raffinose, and glucose 1-phosphate), phosphate, erythritol, threitol, mannitol, campesterol and stigmasterol, and low levels of FFA (palmitic, linoleic, linolenic and stearic acids), glycerol, sorbitol, β-sitosterol, threono-1,4-lactone. The left-hand part harbors accessions with the opposite values of the above-listed compounds, the upper part with minimal amounts of sorbose and fructose, and the bottom part with the highest levels of these two sugars. Thus, the accessions reproduced in 2013 clustered in the section with high amounts of most of the sugars, while those of 2012 in the FFA section. Adverse growing condition (2012) provoked the accumulation of glycerol, sorbitol and β-sitosterol, whereas optimal conditions for plant growth and development (2013) favored erythritol, threitol, mannitol, campesterol and stigmasterol. The wild species L. vernus, L. linifolius and L. tuberosus showed intermediate levels of most sugars, polyols and FFA, and low values of fructose and sorbose. The accessions of L. sylvestris differed from other wild forms in the higher fructose and sorbose content. Many indicators in L. latifolius were close to those in L. sylvestris. The cultivated L. sativus demonstrated the richest polymorphism of all the studied characters. Most of the cultivars representing L. sativus concentrated in the section of intermediate and high levels of fructose and sorbose, but no species-specific groups were observed for the other biochemical characters: the accessions scattered across the graph in accordance with individual features of their genotypes and their normal responses to weather conditions.

Factors F3–F10 highlighted heterogeneity of the accessions in each of the principal components (Suppl. Material 3). Maximums of factor loadings for most of the factors were recorded in the accessions of L. sativus (k-34 and k-900), L. linifolius (N-597422), and L. vernus (N-591179 and N-593953).

According to Chavan (1998), the green biomass of peavines is characterized by a fairy high content of sugars. Within the plant, sugars play the role of osmoprotectants and stabilizers of membranes and proteins, including the same under the impact of low temperatures. Therefore, accessions with high sugar content, especially as far as oligosaccharides and raffinose are concerned, are more resistant to abiotic environmental stressors. Oligosaccharides are accumulated by a plant as a response to the cold stress (Krasenski, Jonak, 2012; Moreno et al., 2018). In our experiment, the growth of the oligosaccharide content was observed in the year with lower temperatures, which confirmed the above-mentioned. The highest levels of raffinose were observed in L. sativus, so its accessions may be recommended to breeders to develop stress-adaptable and cold-tolerant cultivars.

Some researchers reported that if the raffinose content in food or feed exceeded 0.4 % dry weight it may cause dyspeptic disorders (Muzquiz et al., 2012). In our experiment, its percentage never exceeded this level, so the green biomass of all studied peavine species may be used as animal feed.

Mannitol, arabinitol, sorbitol, galactinol, dulcitol, erythritol and other polyols improve adaptive properties of a plant against salt, water and temperature stresses. Accessions with a high content of these compounds may be identified as potentially resistant to unfavorable environmental factors (Tibbett et al., 2002; Majumder, Biswas, 2006; Dong et al., 2013; Baudier et al., 2014; Zhou et al., 2014; Patel, Williamson, 2016; Moreno et al., 2018). According to our data, the content of glycerol and sorbitol in peavine green biomass was higher under cold and dry conditions in 2012 (23 and 56 c. u., respectively), which confirmed the results of the authors referred to above. The highest levels of glycerol and sorbitol were observed in L. sativus.

Inositol, its isomers and derivatives, including phosphate ones (methyl-inositol, chiro-inositol, myo-inositol, ononitol, and myo-inositol 2-phosphate) participate in the cell membrane biosynthesis and plant growth regulation, are incorporated into the composition of the cell’s phosphate ‘depot’, and appear among the antistress factors of plant protection (osmolites) (Dong et al., 2013). In this research, when the plants developed under cold dry conditions, their mean values of ononitol were higher than in an optimal environment (505 and 245 c. u., respectively). The same situation was observed with the levels of mannitol, xylitol and erythritol in the green biomass of L. sativus: under unfavorable conditions their content increased. The maximums of methyl-inositol, chiroinositol, and myo-inositol 2-phosphate were identified in L. sativus (200, 40 and 0.5 c. u., respectively), while myo-inositol and ononitol maximums in L. linifolius (212 and 2088 c. u., respectively). On the whole, high values in the polyol content were recorded for all Lathyrus spp., which explains their resistance to abiotic and biotic stressors.

Phytosterols play an important role in plant growth and development, because they are precursors of phytohormones (Beebe, Turgeon, 1992; Deng et al., 2016; Valitova et al., 2016). According to published data, the main phytosterol in plants is β-sitosterol, which coincides with our results: β-sitosterol accounted for 86.5 % of the total content of identified sterols. In our experiment, the total phytosterol content in the peavine biomass was 39 c. u.

Among FFA on the surface of L. sativus seeds, palmitoleic and palmitic acids are prevailing, followed in descending order by stearic, myristic, oleic, arachidic, capric, behenic, linoleic and linolenic acids (Adhikary et al., 2016). In this study, the major share of identified FFA also went to palmitic, linoleic, linolenic, oleic and stearic acids. The presence of the mentioned FFA in green biomass characterizes peavines as highly nutritional fodder plants promising for cultivation.

Thus, over 300 compounds have been identified in the green biomass of Lathyrus spp. (Solovyeva et al., 2019); more than 60 of them are described in this publication. The values of a majority of the analyzed biochemical substances demonstrated a wide range of variability. Their amounts significantly varied across different genotypes, species, and years of testing. The studied accessions showed high intra- and interspecific polymorphisms, both in the quantitative content and qualitative composition of the identified compounds.

Wild species (L. vernus, L. linifolius and L. tuberosus) had medium values in the content of most sugars, polyols and FFA, and low levels of fructose and sorbose. As far as their sugar content is concerned, L. sylvestris and L. latifolius occupied an intermediate position between the abovementioned wild species and L. sativus. The cultivated L. sativus stood out for the highest amount of sugars in its green biomass, L. linifolius for ononitol, myo-inositol and glycerol 3-phosphate, while L. vernus for MAG and methylpentofuranoside.

The comparative analysis helped to identify accessions suitable for further profound investigations into sugars, polyols, FFA, phytosterols, etc. It is especially valuable for future researching, because these compounds are indicators of green biomass quality and resistance to unfavorable environmental factors. The identified accessions can be used to produce both highly nutritional and resistant cultivars of Lathyrus spp. Considering the latest achievements in genomics, such accessions may be regarded as quality and resistance sources not only for peavines but for other crops as well.

## Conclusion

Considerable polymorphism of biochemical characters has been disclosed in wild and cultivated Lathyrus spp. The results obtained attest to the high potential of the studied species for contemporary agricultural production and new breeding trends.

Introducing L. sativus, L. sylvestris, L. vernus, L. linifolius, L. latifolius and L. tuberosus into animal feed production would expand the assortment of the exploited fodder crops. Due to their resistance to abiotic stressors and the disclosed nutritional value, Lathyrus spp. could play an important role in food security maintenance in areas with unpredictable weather conditions.

## Conflict of interest

The authors declare no conflict of interest.
